# Permanent Investments

**Published:** 1864-12

**Authors:** 


(FROM THE "INDEPENDENT.")

### PERMANENT INVESTMENTS.

INVESTING in champagne at $2 a bottle—an acre of good Government land costs $1.25.

Investing in tobacco and cigars, daily, one year, $50—seven barrels of good flour will cost $49.

Investing in "drinks" one year, $100—$100 will pay for ten daily and fifteen monthly periodicals.

Investing in theatrical amusements one year, $200—$200 will purchase an excellent library.

Investing in a fast horse, $500—400 acres of good wild land costs $500.

Investing in a yacht, including bettings and drinkings for the season, $5000—$5000 will buy a good improved country farm.

Panics, hard times, loss of time, red faces, bad temper, poor health, ruin of character, misery, starvation, death, and a terrible future may be avoided by looking at the above square in the face.

Reader, put on your spectacles, take a look, and tell us what *you* think on the subject; and, if a father, ask your boys what *they* think.

A majority of "financiers," in making calculations for the future, watch the importations, exports of specie, the ups and downs of stocks, and the movements of the Wall-street Bulls and Bears. All that is very well, but let them at the same time estimate the loss of gold in the maelstrom of extravagance.

